# Universal Decentralized Cord Blood TSH Screening Should Be Offered as Routine Delivery Care in Limited-Resource Settings

**DOI:** 10.3390/ijns11040105

**Published:** 2025-11-14

**Authors:** Nitash Zwaveling-Soonawala, Anju Virmani, Aman B. Pulungan, Joseph Haddad, Sirisha Kusuma Boddu, Feyza Darendeliler, A. S. Paul van Trotsenburg

**Affiliations:** 1 Department of Pediatric Endocrinology, Emma Children’s Hospital, Amsterdam UMC, University of Amsterdam, 1105AZ Amsterdam, The Netherlands; n.zwaveling@amsterdamumc.nl (N.Z.-S.); a.s.vantrotsenburg@amsterdamumc.nl (A.S.P.v.T.); 2 Amsterdam Gastroenterology Endocrinology Metabolism, Amsterdam UMC, University of Amsterdam, 1105AZ Amsterdam, The Netherlands; 3 Endocrinology Division, Department of Pediatrics, Max Smart Super Specialty Hospital, Saket, New Delhi 110017, India; 4 Department of Endocrinology, Madhukar Rainbow Children’s Hospital, New Delhi 110017, India; 5 Department of Child Health, Faculty of Medicine, Universitas Indonesia, Jakarta 10430, Indonesia; amanpulungan@mac.com; 6 International Pediatric Association, 9804 Voss Road, Marengo, IL 60152, USA; profhadad@gmail.com; 7 Department of Pediatric Endocrinology and Diabetes, Rainbow Children’s Hospital, Hyderabad 500032, India; sirisuma@gmail.com; 8 Emeritus, Pediatric Endocrinology, Faculty of Medicine, Istanbul University, Istanbul 34093, Turkey; feyzadarendeliler@gmail.com

**Keywords:** congenital hypothyroidism, newborn screening, cord blood, TSH

## Abstract

Newborn screening (NBS) for congenital hypothyroidism (CH) facilitates early diagnosis and treatment and prevents permanent intellectual disability. Sadly, 50 years after the first introduction of NBS for CH, only 29.6% of newborns worldwide are screened. Africa and Asia, the continents with the highest birth rates, have very limited screening coverage. Most NBS programs measure TSH in a dried-blood spot taken from a heel-prick on a filter paper after 24 to 72 h of life. Implementing national NBS programs is logistically complex and expensive, requiring parental consent, specialized laboratories, and excellent infrastructure. In limited-resource settings, introducing such a complex program is often impossible. We propose universal decentralized cord blood TSH screening, offered as routine delivery care for all newborns in limited-resource settings. TSH measurement may be performed by local laboratories using widely available, inexpensive radioimmunoassay kits, with the report available within a few hours. Since the TSH report would be available before discharge, suitable clinical decision making would be possible, with a minimal need for recall, thus minimizing the parental, medical, and financial burden and improving developmental outcomes. The most important requirement is to change to a grassroots approach, with the education of obstetricians and pediatricians worldwide to perform routine cord blood TSH and make sure the TSH result is available before the baby is discharged.

## 1. Introduction

The value of newborn screening (NBS) for congenital hypothyroidism (CH) is undisputed. Due to the crucial importance of thyroid hormone for the developing brain, untreated CH may cause irreversible brain damage and, with that, severe lifelong mental and motor disabilities [1]. Early treatment of CH with levothyroxine, when started as early as possible, preferably within the first two weeks of life, has proven to be effective in preventing such neurodevelopmental sequelae [1,2,3].

Since most newborns with CH are asymptomatic at birth, a clinical diagnosis is usually delayed. Hence, to facilitate early diagnosis and treatment, NBS for CH was started. The first introduction of NBS for CH was in Quebec, Canada, in 1974; since then, many countries have implemented national NBS programs for CH [4,5,6]. From 1995 to 2007, the International Atomic Energy Agency (IAEA) spent an estimated 6.7 million USD to help set up NBS programs for CH in 29 countries [7]. Numerous follow-up studies have proven that NBS for CH is effective in enabling early detection and treatment of CH and that children who are treated early have excellent neurodevelopmental outcomes [2,3,8]. It is also highly cost-effective, especially in low- and middle-income countries (LMICs) [9]. Therefore, international guidelines recommend that all newborns must receive NBS for CH, and that treatment should be initiated early, at least within the first two weeks of life, to ensure normal brain development [1,10,11].

Fifty years after the first introduction of NBS for CH, a recent study reported that worldwide, only 29.6% of newborns actually receive screening for CH [12]. Ten years earlier, in 2014, a similar study by Ford and LaFranchi had almost identical results [13]. It is tragic that worldwide, more than 70% of newborns are still not screened for CH and that this situation has not changed in the last decade. National programs continue to struggle due to political and financial reasons; e.g., many programs ceased when IAEA funding stopped in 2007 [7].

An annual global birth rate of 132 million [14] means approximately 92 million newborns are not screened for CH. The reported prevalence of CH is between 1:3000 and 1:2000 newborns [1]. But in many regions, this may be higher. The Indian Council for Medical Research Task Force reported the overall prevalence as 1 in 722 births and after excluding transient hypothyroidism, the prevalence was calculated to be 1 in 1130 [15]. If CH has an incidence of approximately 1 in 1000–2000 newborns and more than 70% newborns are not screened, an annual estimated 46,000–92,000 cases of CH are going untreated, with probable detrimental neurodevelopmental sequelae, imposing a lifelong burden on the family. This is especially sad since levothyroxine treatment is inexpensive, easy to administer, and widely available, including in LMICs.

**Figure 1:**
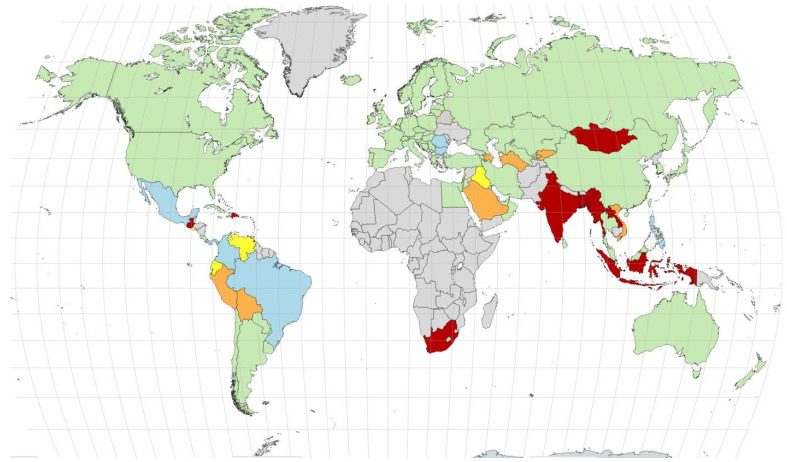
Coverage rates for neonatal screening worldwide in 2024. Colors indicate coverage rates (green indicates coverage rates ≥ 90%, light blue indicates coverage rates between 75 and 90%, yellow indicates coverage rates between 50 and 75%, orange indicates coverage rates between 25 and 50, and red indicates coverage rates < 25%). Countries marked in gray either have no NBS program or no data (the exception is Greenland, which is screened under the Danish NBS program with >99% coverage). Figure originally published in reference [12].

Figure 1 depicts worldwide screening rates for CH [12]. CH screening is mainly performed in high- and middle-income countries. Africa and Asia, the continents with the highest birth rate, have very limited screening coverage [12].

In Africa, the only national NBS programs for CH have been reported in Egypt and South Africa [12]. Egypt has a national NBS program for CH reaching over 90% of newborns. In South Africa, CH screening is mainly offered in private health care facilities, while in the public system, the screening coverage is only around 4%. Other countries in Africa have had pilot studies to screen for CH but have not been able to expand and maintain these initiatives, often due to a lack of financial and political support. Also, geographic challenges complicate the accessibility of NBS programs [16].

In Sub-Saharan Africa, it is more important to screen for sickle cell disease (SCD) because of its high incidence and the morbidity and mortality associated with this condition. While various initiatives to implement NBS for SCD in Sub-Saharan Africa have been implemented, very few countries have a national screening program [16]. It might be efficient to incorporate CH screening into existing or new NBS programs for SCD [17].

In Asia, some countries do have national NBS programs for CH. China, with the world’s second highest annual birth rate of 17 million, has a longstanding national NBS program with a coverage of around 98%. Japan has had NBS for CH since 1979 with almost 100% coverage [12]. In the last decade, several countries in Asia have shown substantial improvements in screening coverage rates. For example, the rate in Sri Lanka rose from 2.8 to 92%, that in Vietnam increased from 7 to 38.5% [12,13], and that in Indonesia rose from 1 to 28% in 2023 and is increasing rapidly [7]. In Thailand, the NBS-for-CH program initiated in 1992 had achieved more than 94% coverage by 2015. Several developing nations in South America have reported similar successes. In Nicaragua, cord blood-based CH screening has reached 80% coverage. Decentralized through more than 175 laboratories, Cuba’s national NBS program covers more than 99% of the population, while Mexico, through coordinated efforts across different components of its complex health care system, had achieved over 80% NBS coverage by 2015 [18]. This is in vast contrast with India, the country with the world’s highest birth rate of 24 million per annum, where approximately only 3% of newborns receive screening for CH [12]. Despite several pilot studies in the last few decades, only three states in India have been able to initiate and sustain a NBS program [19].

## 2. Newborn Screening Strategies

Various strategies to screen for CH are being used (measurement of TSH (only), TSH + thyroxine (T4) or Free T4 (FT4), or T4-reflex TSH-reflex thyroxine binding globulin (TBG)), but the most used method is a single TSH measurement in a dried-blood spot (DBS) collected by a heel-prick on a filter paper card after 24 to 72 h of life. An elevated TSH indicates primary CH, and this strategy enables early detection and initiation of levothyroxine treatment, usually within the first two weeks of life.

It is important to realize that immediately after birth, a physiological TSH surge takes place, driven by exposure to cold during delivery. TSH peaks around 30 min after birth, after which it rapidly declines in the first 24 h and gradually further normalizes in the first 48 to 72 h after birth. Therefore, a blood withdrawal from the infant for TSH measurement within the first 24 to 48 h of life, e.g., at the time of discharge, is within the TSH surge phase. This leads to many false-positive screening results, causing a needless logistic and financial burden on the screening program. So, the windows for reliable CH screening are either cord blood TSH, which is prior to the physiological TSH surge, or postnatal TSH measurement ≥ 48 h after birth.

Most existing NBS programs use TSH measurement in DBS collected after 24 to 72 h of life. An important advantage of such postnatal samples is the possibility to screen for additional conditions such as congenital adrenal hyperplasia and metabolic diseases dependent on feeding, such as phenylketonuria and galactosemia. However, this policy of postnatal sampling imposes complex logistics involving the calling back of infants in case of early discharge and has several other disadvantages, which make universal screening very difficult in developing countries where early discharge is the norm, often within 24 h after birth.

An alternative idea is to change our approach entirely from national programs to decentralized grassroot care, performing cord blood TSH measurement as part of routine delivery care for every newborn, while continuing with existing or future programs of collecting and testing DBS after 24 to 72 h of life in as many babies as possible.

## 3. Obstacles in Implementing NBS in Limited-Resource Settings

Setting up centralized NBS programs in limited-resource settings is very challenging. The ten primary challenges that have previously been identified are planning, leadership, education, medical, technical, and logistical support, policy development, administration, evaluation, and sustainability [20].

Newborn screening with DBS collection on day one to two after birth, i.e., during the period of the thyroid surge, is likely to increase recall rates, thereby leading to significant cost and work for the medical system and inconvenience for the family. A policy of testing after 24 to 72 h of life would mean either calling back newborns or sending a health worker to their homes in the case of the early discharge of the mother and baby, which in developing countries is often within 24 h after birth. It becomes necessary to trace all newborns and convince parents to bring in their seemingly healthy infant for an invasive heel-prick—consent is often refused, more so when expensive tests have to be paid for out of pocket. In addition, this method requires the availability of specialized filter paper cards, special technical skills to apply the blood spot on the filter paper card, and, furthermore, specialized laboratory techniques to extract and measure TSH from these filter paper blood spots. This is usually not possible in local laboratories, which means transporting the samples to centralized specialized laboratories. Subsequently, in the case of an abnormal result, this needs to be communicated to the staff at the birthing center, who in turn need to contact the parents and arrange to perform follow-up testing to confirm the abnormal screening result. Such an approach calls for excellent infrastructure, specialized laboratories, the education of health care workers, and cooperation from the parents of a healthy-looking baby. Support from regional governments and considerable financial outlay are required in order for such centralized screening programs to be successful.

Pulungan and co-workers performed a national cross-sectional survey on experiences and challenges in NBS for CH in Indonesia [21]. This included responses from 423 health care professionals. Major challenges reported included refusal from families to cooperate (39.2%), newborns being discharged within 24 h (38.3%), and limited availability of filter paper (35.9%). In addition, the lack of infrastructure and education of health care workers were mentioned as important obstacles in implementing NBS [21]. In a feasibility study by the Indian Council for Medical Research, heel-prick DBS samples were collected at or after 24 h of age (in the midst of the thyroid surge). Even in project mode, only 73.2% of babies could be sampled by personnel; i.e., over one quarter of babies were missed [22]. The concluding recommendation for the immediate start of a national screening program remains unfulfilled almost a decade later.

## 4. Simplifying Newborn Screening by Universal Decentralized Cord Blood TSH Screening

The logistical complexities of centralized NBS programs for CH, further hampered by the early discharge of mother and baby, have been major obstacles in implementing NBS using DBS after 24 to 72 h of life, resulting in continued low screening rates in Africa and Asia.

To overcome these obstacles, we propose the implementation of universal decentralized cord blood TSH screening in all limited-resource settings. This approach entails collecting a venous cord blood sample from the placental side of the umbilical cord immediately after birth, before the thyroid surge begins, without having to burden the newborn with a heel-prick or making the parents bear considerable additional expenses. Expensive filter paper cards and laboratory techniques are not required. TSH measurement is performed by radioimmunoassay or chemiluminescence kits, which are inexpensive and usually available in local laboratories. The report is usually available in 2 to 24 h. Thus, the birthing center can realistically implement the policy of having the report available before the mother–baby dyad is discharged, obviating the need to make any special efforts to communicate results. In the few newborns whose TSH is raised, the baby is not discharged early, and a confirmatory venous FT4 and TSH after 48 h are advised. The confirmatory report would also be available in a few hours, so any clinical decisions required, i.e., starting thyroxin replacement, could be taken before the baby was discharged. In settings where results take longer, the universality of mobile phones today will help to inform parents and facilitate recall.

The most important requirement is the education of local obstetricians and pediatricians to ensure cord blood collection and TSH testing are performed as a routine part of all deliveries and to make sure the TSH test result is reported early, before the mother and baby are discharged. The cord blood TSH measurement should be incorporated as standard care, just like postnatal vitamin K supplementation. It need not conflict with any pre-existing or planned DBS testing being offered after 24 to 72 h of life to test for multiple conditions. In limited-resource settings, any DBS program is likely to cover a very small proportion of infants, and it should be continued while aiming for 100% coverage with universal cord blood TSH testing.

TSH can be reliably measured in cord blood and has been proven to be effective in detecting CH. In fact, some high-income countries such as Finland and Singapore have longstanding NBS programs using cord blood TSH measurement [23,24]. In these countries, cord blood TSH measurement is followed by a second postnatal measurement for other conditions. Compared to DBS testing, cord blood TSH measurement enables even earlier diagnosis and treatment of infants with CH. A recent study from Finland showed that such a very early treatment start (age 3.6 days vs. 4.8 days) may improve cognitive outcomes [25].

An additional advantage of cord blood compared to DBS on filter paper is the opportunity to also measure the FT4 concentration. FT4 measurement is technically difficult and routinely not possible in DBS samples. In the case of a mildly elevated TSH, an additional FT4 measurement performed on the same cord blood sample reduces unnecessary recalls. Finland and Singapore have incorporated additional FT4 measurement into their existing NBS program based on cord blood TSH screening [23,24].

The cord blood TSH cutoff determines the number of recalls with a higher cutoff, leading to a lower number of false positives and hence a lower recall rate. In Singapore, infants with a cord blood TSH > 25 mIU/L are recalled by day 3–4 for further evaluation with a venous TSH and FT4. Using this TSH cutoff, the recall rate in Singapore is reported to be 1% [24]. In Finland, NBS is managed by central hospitals and regional laboratories, and TSH cutoffs for confirmatory testing vary between 20–40 mIU/L and 25–40 mIU/L. A Finnish study using a cutoff >20 mIU/L reported a 0.76% false-positive rate [23].

A recent report of a two-year cohort of newborns at a hospital in New Delhi, where universal newborn screening using cord blood was started in 2006, with additional measurement of FT4 from 2014 onwards, suggested simplifying the approach and taking advantage of the short turnaround time for TSH testing, to make cord blood screening more practical [25]. A cutoff of TSH > 20 mIU/L led to a recall rate of 2.7%, with 8.6% no shows, with a cutoff of TSH > 40 mIU/L reducing the recall rate to 0.09%. In order to reduce the number of recall measurements, a simplified strategy of “retest and recall” using three cord blood TSH categories was proposed: (a) TSH < 20 mIU/L: normal, discharge; (b) TSH 20–40 mIU/L: test cord blood FT4; if <0.7 ng/dL, do not discharge early, and perform confirmatory venous TSH and FT4 at 72 h age (before discharge); (c) TSH > 40 mIU/L: do not discharge early, and perform confirmatory venous TSH and FT4 at 72 h (before discharge). If hypothyroidism is confirmed in the venous sample, levothyroxine treatment can be started within a few days after birth [26]. An example of this was a baby whose cord blood TSH was >1000 mIU/L: a confirmatory venous sample the next morning showed an equally high TSH, with low fT4, while a Tc99 thyroid scan showed a lingual thyroid. Replacement with levothyroxine could be started within 24 h after birth, and the now 5-year-old child has normal growth and an above-average intelligence [27].

The strategy of universal decentralized cord blood TSH measurement as part of routine delivery care was discussed during a recent webinar organized by the International Pediatric Association (IPA), with a strong case built for implementing it in limited-resource settings [28]. Once screening begins, the numbers are not so intimidating. Let us take the example of India with an annual birth rate is 25 million. The early preterm rate is 6–7%, and overall institutional deliveries have increased to 88.6%, and even 100% in some states like Kerala and Goa [29]. An analysis of cord blood TSH screening over 16 years’ experience of 164,163 neonates found 2352 (1.4%) had TSH > 25 mIU/L and were recalled for confirmatory testing [30]. Thus, of 1000 babies born, 880 would be delivered in a birthing center, of whom 870 babies would have normal TSH, 10 would need confirmatory testing, and 1 would need treatment. Of the total 880, perhaps 50–55 may be early preterm babies, who would need medical attention anyway. Thus, the total extra burden on the medical system would entail perhaps delaying discharge and performing confirmatory testing in 10 out of every 1000 newborns.

We acknowledge that a local or nearby laboratory able to measure TSH is essential in order for a program using cord blood TSH measurement to be implemented. This is not difficult to achieve over time, as even rural clinics would have some arrangements for laboratory support, e.g., for testing hemoglobin or other basic parameters, and a laboratory would be willing to organize any test for which a steady demand exists. In addition, the quality of testing and the turnaround time would also improve with regular testing. Initially, we may not be able to reach all newborns, especially in case of home deliveries and in remote rural areas, but this strategy would certainly result in a substantial increase from the current low screening rates. With time, a further increase in screening rates and awareness of the importance of newborn screening would be likely. Universal decentralized cord blood TSH measurement can also be used in countries that are currently screening by DBS, where the time of diagnosis is beyond 15 days of age.

In conclusion, at present, worldwide, 70% of newborns are not screened for CH in spite of its undoubtedly favorable cost–benefit ratio. Despite various initiatives, implementing NBS in limited-resource settings has proven to be a logistic nightmare. Lack of infrastructure, specialized laboratory facilities, and high costs, together with early discharge of mother and baby complicate NBS programs using DBS collected after 24 to 72 h of life. High false-positive rates hinder DBS on day one to two due to the TSH surge. TSH measurement performed locally is as inexpensive as levothyroxine treatment. We therefore propose the promotion of universal decentralized cord blood TSH measurement, to be offered as routine delivery care for all newborns in limited-resource settings, including LMICs. This approach, which simply requires widespread awareness among gynecologists, pediatricians, and other health care workers attending deliveries, can achieve substantially higher screening rates.

## Data Availability

Data sharing is not applicable since no new data were created or analyzed in this commentary.

## Abbreviations

The following abbreviations are used in this manuscript:

CH	Congenital hypothyroidism
DBS	Dried-blood spot
FT4	Free thyroxine
IAEA	International Atomic Energy Agency
IPA	International Pediatric Association
LMICs	Low- and middle-income countries
NBS	Newborn screening
SCD	Sickle cell disease
T4	Thyroxine
TBG	Thyroid-binding globulin
TSH	Thyroid-stimulating hormone
